# Two types of dominant male cichlid fish: behavioral and hormonal characteristics

**DOI:** 10.1242/bio.017640

**Published:** 2016-07-18

**Authors:** Rosa M. Alcazar, Lisa Becker, Austin T. Hilliard, Kai R. Kent, Russell D. Fernald

**Affiliations:** Biology Department and Neuroscience Institute, Stanford University, Stanford, CA 94305, USA

**Keywords:** Cichlids, Behavior, Aggression, Stress, Endocrine responses, HPI/A axis

## Abstract

Male African cichlid fish, *Astatotilapia burtoni*, have been classified as dominant or subordinate, each with unique behavioral and endocrine profiles. Here we characterize two distinct subclasses of dominant males based on types of aggressive behavior: (1) males that display escalating levels of aggression and court females while they establish a territory, and (2) males that display a stable level of aggression and delay courting females until they have established a territory. To profile differences in their approach to a challenge, we used an intruder assay. In every case, there was a male-male confrontation between the resident dominant male and the intruder, with the intruder quickly taking a subordinate role. However, we found that dominant males with escalating aggression spent measurably more time attacking subordinates than did dominant males with stable aggression that instead increased their attention toward the females in their tank. There was no difference in the behavior of intruders exposed to either type of dominant male, suggesting that escalating aggression is an intrinsic characteristic of some dominant males and is not elicited by the behavior of their challengers. Male behavior during the first 15 min of establishing a territory predicts their aggressive class. These two types of dominant males also showed distinctive physiological characteristics. After the intruder assay, males with escalating aggression had elevated levels of 11-ketotestosterone (11-KT), testosterone, estradiol, and cortisol, while those with stable aggression did not. These observations show that the same stimulus can elicit different behavioral and endocrine responses among *A. burtoni* dominant males that characterize them as either escalating or stable aggressive types. Our ability to identify which individuals within a population have escalating levels of aggressive responses versus those which have stable levels of aggressive responses when exposed to the same stimulus, offers a potentially powerful model for investigating the underlying molecular mechanisms that modulate aggressive behavior.

## Abstract (precis)

**Summary:** We discover a subclass of dominant male in *A. burtoni* with escalating aggression that displays distinct behavioral and endocrine profiles, which may serve as a model to investigate maladaptive aggressive behavior.

## INTRODUCTION

Individuals behave differently even under identical social circumstances. Darwin appreciated individual variation, and famously wrote: “No one supposes that all the individuals of the same species are cast from the very same mould” (Darwin, 1859). Terms currently being used to study variation in individual behavior include coping styles (Koolhaas et al., 1999), behavioral syndromes (Sih et al., 2004; Bell, 2007) and temperament or personality (Dall et al., 2004; Réale et al., 2007). Individual differences in behavior have been identified in many vertebrates including fish (Huntingford, 1976), lizards (Cote and Clobert, 2007), birds (Groothuis and Carere, 2005) and rodents (Koolhaas et al., 1999). In the last few decades, many behavioral ecologists and evolutionary biologists have embraced analysis of intraspecific differences in behavior (Dall et al., 2004; Dingemanse and Réale, 2005), intraspecific adaptability across environmental and social contexts (Dingemanse et al., 2010) and have devised methods to measure phenotypic plasticity (Bell, 2007; Martin et al., 2011). These studies may help us understand the underlying genetic, endocrine and experiential causes for personality differences in other species including humans (Sih et al., 2004).

Clinical neuroscientists seeking insights into the underlying causes for the variation in resilience to trauma and susceptibility to maladaptive behavior in individuals, hoping to find therapies to modulate behavioral responses may find individual differences useful guides in these endeavors (Yehuda and Antelman, 1993; Yehuda and LeDoux, 2007). However, most basic research in behavioral neurosciences concentrates on a few model systems with paradigms that challenge animals with ecologically irrelevant stressors (Koolhaas et al., 2006). Challenging an animal's stress-physiology within its normal allostatic range will help identify the underlying causes for individual differences in behavior and adaptability (Koolhaas et al., 1999, 2006).

*Astatotilapia burtoni* males can undergo remarkable changes in behavior and physiology in response to changes in their social environment. In their natural habitat, Lake Tanganyika in east Africa, the species has a lek-like social system where some males compete for and defend territories used to solicit females for spawning. Changes in substrate cover and predation create a turnover of territory ownership, decided by male-male fights with winners controlling available space. Food is limited and only a fraction of males (10-30%) maintain territories where females come to feed and spawn (Fernald and Hirata, 1977a,b). Consequently, the initiation, escalation, and attenuation of aggressive behaviors are central components of male reproductive success. Which individuals succeed is determined by the combination of available resources and the individual's willingness to compete and fight for those resources.

Here we characterize the individual differences of this naturally occurring behavior in dominant *A. burtoni* males.

This study had two parts. First, using data collected for previous studies (Alcazar et al., 2014; Brawand et al., 2014), we did a *post hoc* analysis of variability in aggressiveness among ∼300 dominant males. These data suggested two types of dominant *A. burtoni* males coexist in our laboratory population. Second, we used the newly defined classes of dominant males to determine differences in behavior when establishing, maintaining and defending a territory.

Our results reveal that among males classified as dominant, there are two subclasses with distinct behavioral and endocrine profiles. The group we call escalating aggressive is a hyper-aggressive type that is progressively more aggressive over time. Our findings demonstrate that with our assay we can predict, by quantifying early behavior when first establishing a territory, which individuals will develop escalating aggression. The study offers a new behavioral assay that can identify individuals susceptible to escalating aggression and may serve as a new assay to evaluate methods for modulating aggressive behavior.

## RESULTS

### Some dominant males escalate their aggression over time

We created ∼300 dominant-subordinate pairs (dyads) by pairing males in the presence of three females (see Materials and Methods) and recorded differences in the aggressive behaviors among the dominant males (Table 1). We found that: (1) 45% of dominant males did not inflict damage on any other fish; (2) 9% inflicted damage on one other fish but modulated their aggression towards a second opponent and females; and (3) 46% inflicted damage on both opponents and females; that is, they damaged two or more fish. To discover if the time spent in the dyad was the cause of increased aggression, we measured how many days each dominant male spent in the dyad before we found evidence of fish damage (latency to first damage, LFD). Interestingly, the median LFD for animals that damaged only one fish was 19 days (*n*=29) while the median for those that damaged 2-5 ranged from 7 to 11 days. That is, those that damaged multiple fish started to inflict damage in half the time (2+ damage, 9 days, *n*=144; 1 damage, 19 days, *n*=29; Mann–Whitney *P*=0.0003). We also found a significant negative correlation between total number of fish damaged and LFD. That is, the faster a given dominant male damaged the first fish, the more fish he would ultimately damage during the course of the dyad assay (Pearson correlation=−0.26, *P*=0.00034). Thus, the amount of damage/injury inflicted on other animals was not a consequence of time spent in a dyad, but was characteristic of the males with the greatest, and earliest to manifest, aggressive tendencies.

**Table 1.**
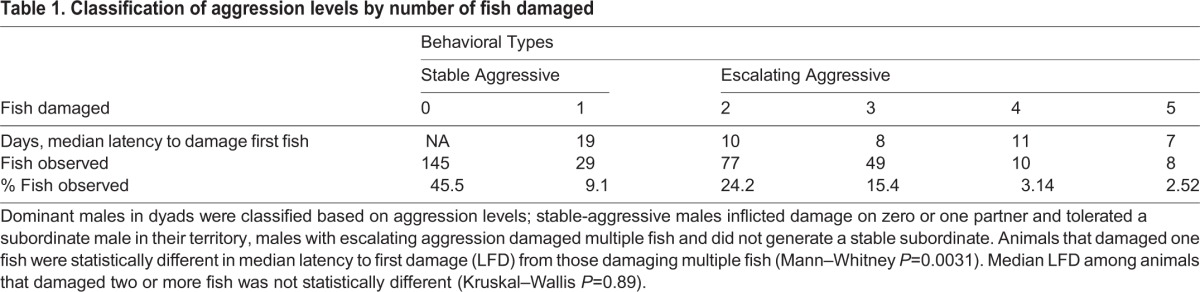
**Classification of aggression levels by number of fish damaged**

To determine if dominant males that damaged multiple opponents escalated their aggression over time we compared the LFD to the latency to damage a second male (*n*=65). The median LFD was 10 days, while the latency to damaging the second opponent was 2 days (*P*<0.0001, Wilcoxon signed-ranks). Thus, males that damaged at least two opponents were quicker to damage the second. Based on these data, we classify dominant fish into two broad groups: those with stable aggression, (males that damaged zero or one fish), and those with escalating aggression (males that damaged two or more fish).

**Fig. 1.**
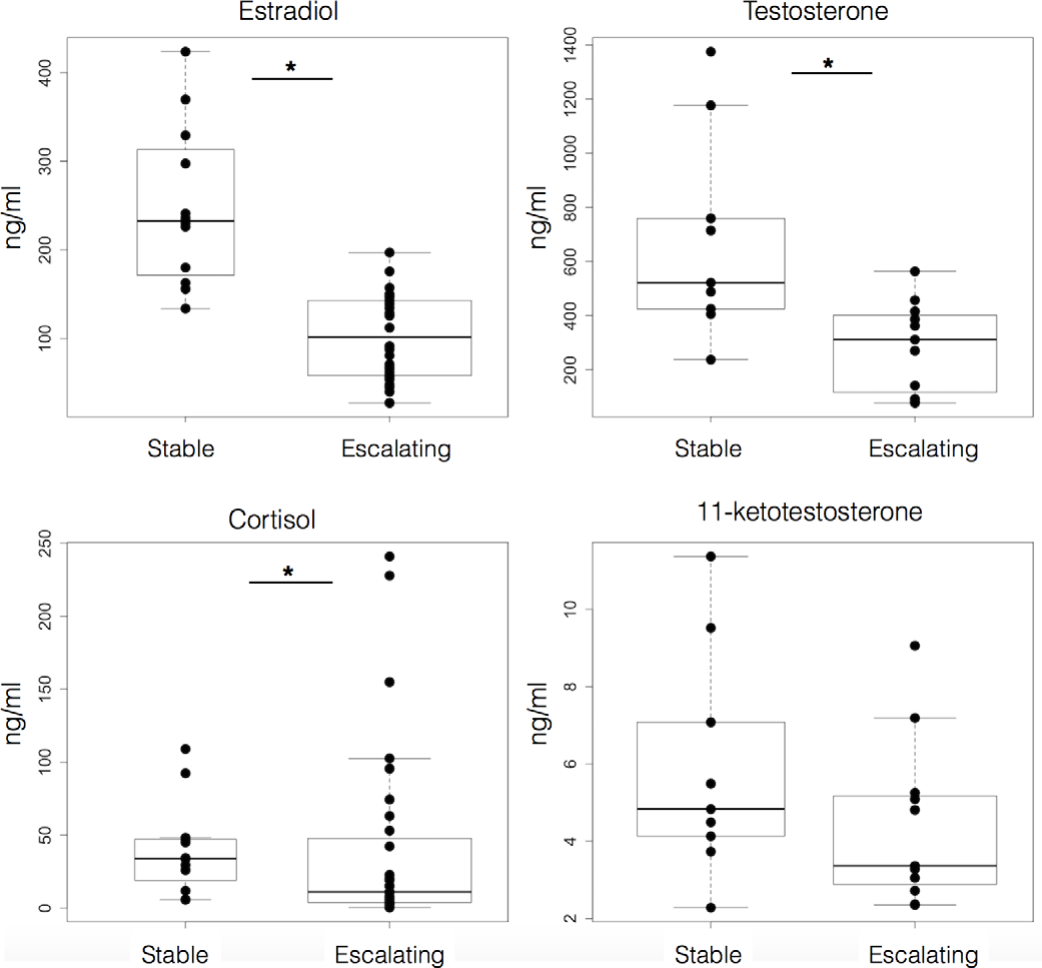
**Males classified as escalating aggressive had lower levels of circulating steroids than stable aggressive types.** Circulating steroid hormones titers after the dyad show males in the escalating aggression category had lower levels of estradiol, cortisol, testosterone but not 11-ketotestosterone. Data are plotted as concentration (ng/ml). Top and bottom of boxes represent the first and third quartiles, respectively; whiskers extend to the most extreme data points no more than 1.5 times the interquartile range from the box; and horizontal lines within the boxes represent group medians. Asterisks indicate a statistically significant difference (*P<0.05, Mann–Whitney).

To place these behavioral differences in a physiological context we measured circulating levels of the steroid hormones estradiol, testosterone, cortisol, and 11-KT among these males at the end of the dyad assay (Fig. 1). Males with escalating aggression had lower titers for estradiol (Mann–Whitney, *P*<0.0001; stable-agg *n*=12; escalating-agg *n*=34), testosterone (*P*=0.006; stable-agg *n*=9; escalating-agg, *n*=11) and cortisol (*P*=0.0189; stable-agg *n*=14; escalating-agg, *n*=22) but not for 11-KT (*P*=0.259; stable-agg *n*=9; escalating-agg, *n*=11), compared to fish with stable aggression.

### Alternative explanations for the dominant male subtypes

Dominant males have larger testes than subordinates relative to their body weight, as measured by calculating the gonadosomatic index (GSI; ratio of gonad weight to body weight ×100). We compared GSIs between escalating and stable aggressive males and found no difference. Likewise, there was no difference in age, body length, or body weight between the two types (*P*>0.05, Wilcoxon rank sum). All dyad observations were made in test tanks of essentially the same size (±0.5 liter), and there was no correlation between tank identity and aggressivity (*P*>0.05, Wilcoxon rank sum). Since a positive correlation between population density and aggressive behavior has been found in many fish studies (Ellis et al., 2002), we asked whether the classification in a dyad test tank was due to the change in fish density between the rearing and dyad tank which was not the case. Animals were transferred from rearing tanks (114 liters) with a population of ∼35 fish (0.3 fish/l) to dyad tanks (30 liters) with a population of five fish (0.16 fish/l) indicating that crowded living conditions could not account for our results. Thus, we concluded that the differences in the aggressive responses of males in the dyads might be the social context (only one territory available and one opponent in the tank), their life-experience, or a genetic predisposition to aggressive behavior.

### Behavior on day 1 predicts future aggressive behavior

**Fig. 2.**
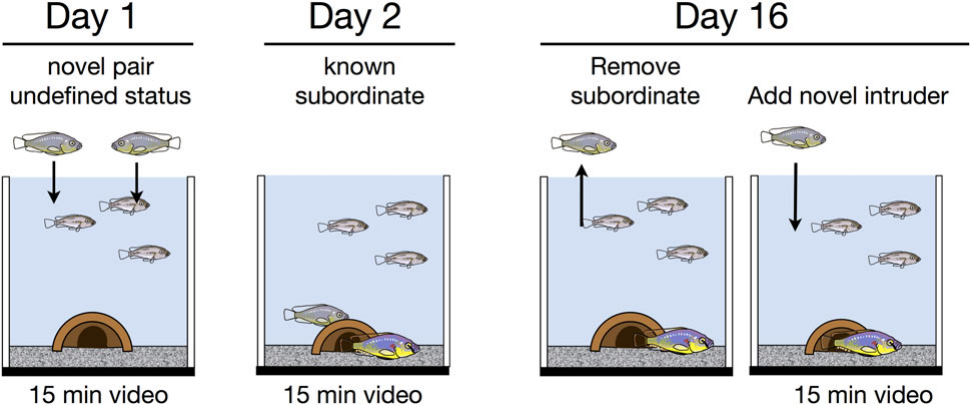
**Social manipulations used to characterize differences in behavior and physiology as a function of aggression.** Animals were moved to test tanks where we recorded the first 15-min of behavior at three time-points (Day 1, Day 2, Day 16). In the first observation period (Day 1) two males were moved to a test tank containing three females. We were agnostic as to which male would become dominant and scored behavior of both males. Twenty-four hours later (Day 2), we scored the behavior of the dominant and subordinate male in each tank. On Day 16 we introduced a novel intruder. To classify dominant males as stable or escalating aggressive, we counted the number of fish damaged in the interval between Day 2 and Day 16.

Based on the above results, we developed a second assay with different animals to determine whether early behavior patterns could predict later aggression. We examined the behavior of a new set of males (see Materials and Methods) on three days (Days 1, 2 and 16; see Fig. 2) over the course of 16 days. We paired males from rearing tanks, controlling for age, size and novelty. Opponents came from different rearing tanks to eliminate effects of experience. We scored male behavior during the first 15 min of Day 1 (a novel environment) and compared the total time spent performing social behaviors (combined male- and female-directed behaviors) and neutral territorial behaviors (non-social territorial behaviors: digging and pot entries). These behavioral data were later compared to the dominant males' subsequent aggressive behavior over the following weeks to test the predictive value of behaviors on Day 1.

**Fig. 3.**
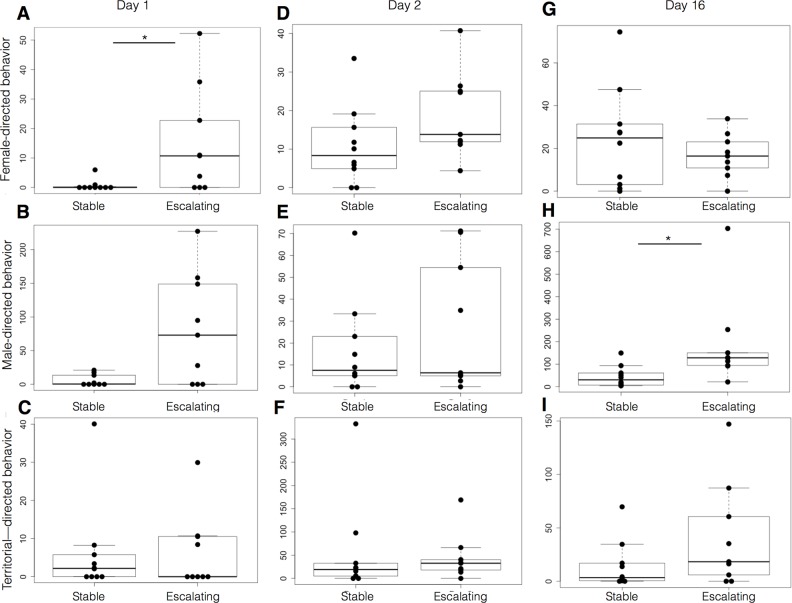
**Males that ultimately display escalating aggression behave differently when placed in different social contexts.** The behavior quantified for 15 min of observation in Days 1, 2 and 16 shows differences between males that were later classified as escalating versus stable aggressive. On Day 1, when males are in a novel territory facing a novel opponent, escalating aggressive males are significantly more proactive: showing more female-directed behaviors prior to establishing a territory (A), a trend in more male-directed behavior (B), and no difference in non-social territorial behaviors (C). On Day 2 the dominant male was familiar with his territory and his opponent. We saw no significant differences in behaviors on this day (D-F). On Day 16, when we introduced a novel intruder, the escalating aggressive type performed more aggressive male-directed behavior toward the challenger (H) but showed no change in female (G) or non-social territorial behavior (I). Behaviors are expressed as time spent (s) engaged in male-directed, female-directed or non-social territorial behaviors. Data are plotted as mean time spent (s). Top and bottom of boxes represent the first and third quartiles, respectively; whiskers extend to the most extreme data points no more than 1.5 times the interquartile range from the box; and horizontal lines within the boxes represent group medians. Asterisks indicate a statistically significant difference (*P<0.05, Mann–Whitney).

We found differences in dominant males on Day 1: males that would later be classified as escalating aggressive (*n*=9) spent significantly more time attending to females (*P*=0.0315, *n*=10; Mann–Whitney; Fig. 3A), a trend for more male-directed behaviors (*P*=0.057; Fig. 3B) but no difference in non-social territorial behaviors (*P*=0.966, Fig. 3C) than males ultimately classified as stable aggressive. In contrast, on Day 2, there were no significant differences in female (*P*=0.09402, Fig. 3D), male (*P*=0.5950, Fig. 3E) or non-social territorial behaviors (*P*=0.4371, Fig. 3F). Thus, males with escalating aggression performed more social behaviors towards both males and females, and performed them earlier, when introduced to a novel environment, than did males with stable aggression.

**Fig. 4.**
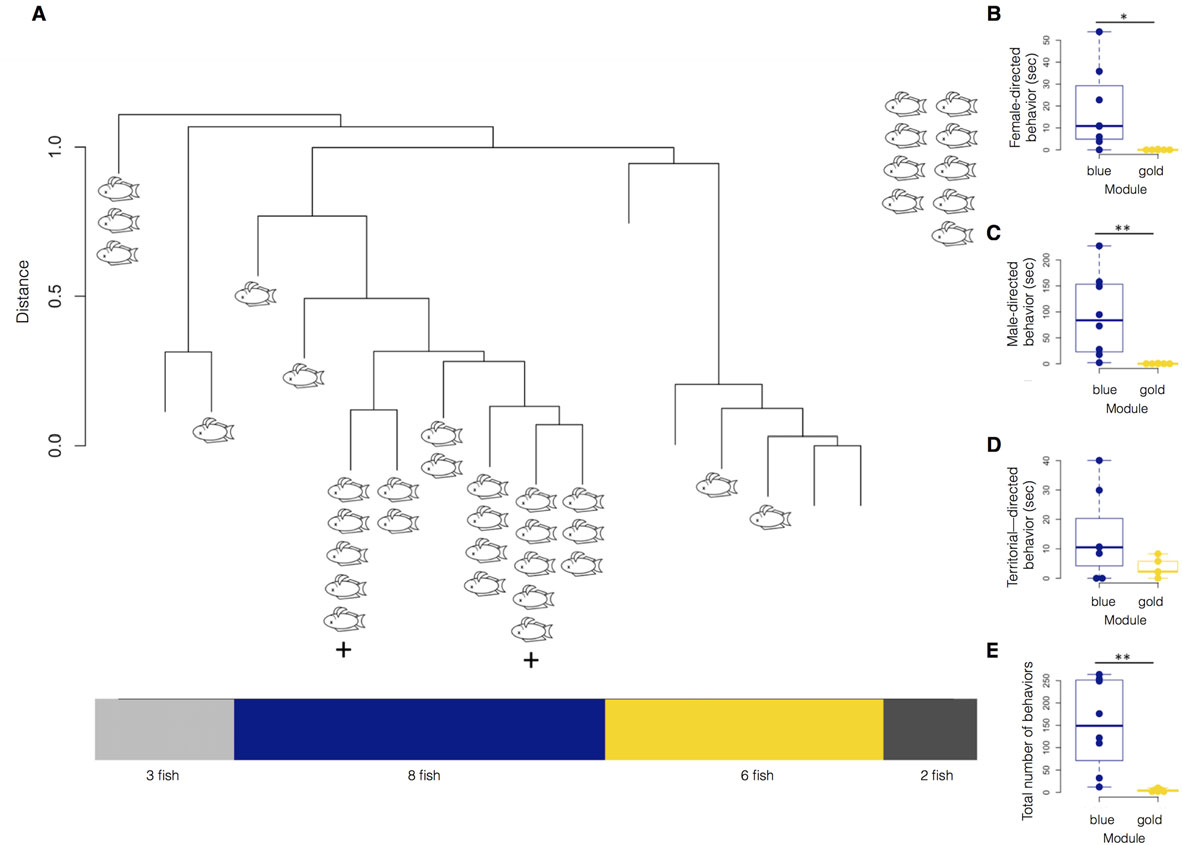
**Results of unsupervised clustering of the fish by the number of incidents of each behavior performed during the first 15 min of Day 1.** (A) The fish cluster into two proper modules, ‘dark blue’ (*n*=8, six escalating aggressive, two stable aggressive) and ‘gold’ (*n*=6, all stable aggressive). Each branch represents one fish (*n*=19). Distance between any two animals is defined as 1 minus the correlations between all their behaviors. Two fish performed 0 behaviors on Day 1, preventing correlations from being computed (both escalating aggressive, shown in dark gray to right), while three fish were unassigned to a module (two stable, one escalating, shown in light gray). Module assignments were significantly similar to later stable/escalating aggressive classification (*P*=0.017, chi squared). (B-E) There are significant differences between the ‘dark blue’ and ‘gold’ module in time spent performing female-directed behaviors (*P*=0.0072, Mann–Whitney) and male-directed behaviors (*P*=0.0019), as well as in the total number of behaviors (*P*=0.0025). There was no significant difference in time spent performing neutral/territorial behaviors (*P*=0.19), although it is worth noting that fish in the ‘gold’ module tended to spend more time performing these behaviors than performing social behaviors.

To test if individuals with escalating aggression displayed early behaviors that could suggest males with escalating aggression had an intrinsic predisposition for their high aggressivity, we tested our classification of males of stable or escalating classes against an unbiased automatic classification. We used an unsupervised hierarchical clustering (Langfelder et al., 2008) that would group our individuals (*n*=19) based on all their behaviors from Day 1. Remarkably, the fish clustered into two modules: one with eight fish (six escalating aggressive and two stable aggressive) and another with six (all stable aggressive, Fig. 4A). When we compared the behaviors of fish based on their module assignments, there were significant differences in time spent performing female-directed behaviors (*P*=0.0072, Mann–Whitney; Fig. 4B), male-directed behaviors (*P*=0.0019, Fig. 4C), and total number of social behaviors (*P*=0.0025, Fig. 4E). There was no significant difference in time spent performing neutral/territorial behaviors (*P*=0.19, Fig. 4D), although it is worth noting that fish in the module with all stable aggression males (in gold) tended to spend more time performing these behaviors than they did performing social behaviors (*P*=0.1056, not shown). In contrast, the module composed predominantly of males with escalating aggression (blue) spent significantly less time performing neutral territorial than social behaviors (*P*=0.02344, not shown). Although the module assignments did not agree 100% with our original classifications based on observations of damage to other fish in the large dataset, the overlap was statistically significant (*P*=0.017, chi squared).

To determine whether the behavior of the dominant males was a response to the behavior or aggressiveness of the subordinates, we compared aggressive behaviors of subordinate males. Since the subordinates performed zero aggressive behaviors and very few behaviors overall, these tests had relatively low statistical power, but we found no differences in the number of approaches towards the dominant males of either class (*P*>0.05, Mann–Whitney).

### A novel environment had a different effect on the behavior of males in the aggressive classes

**Fig. 5.**
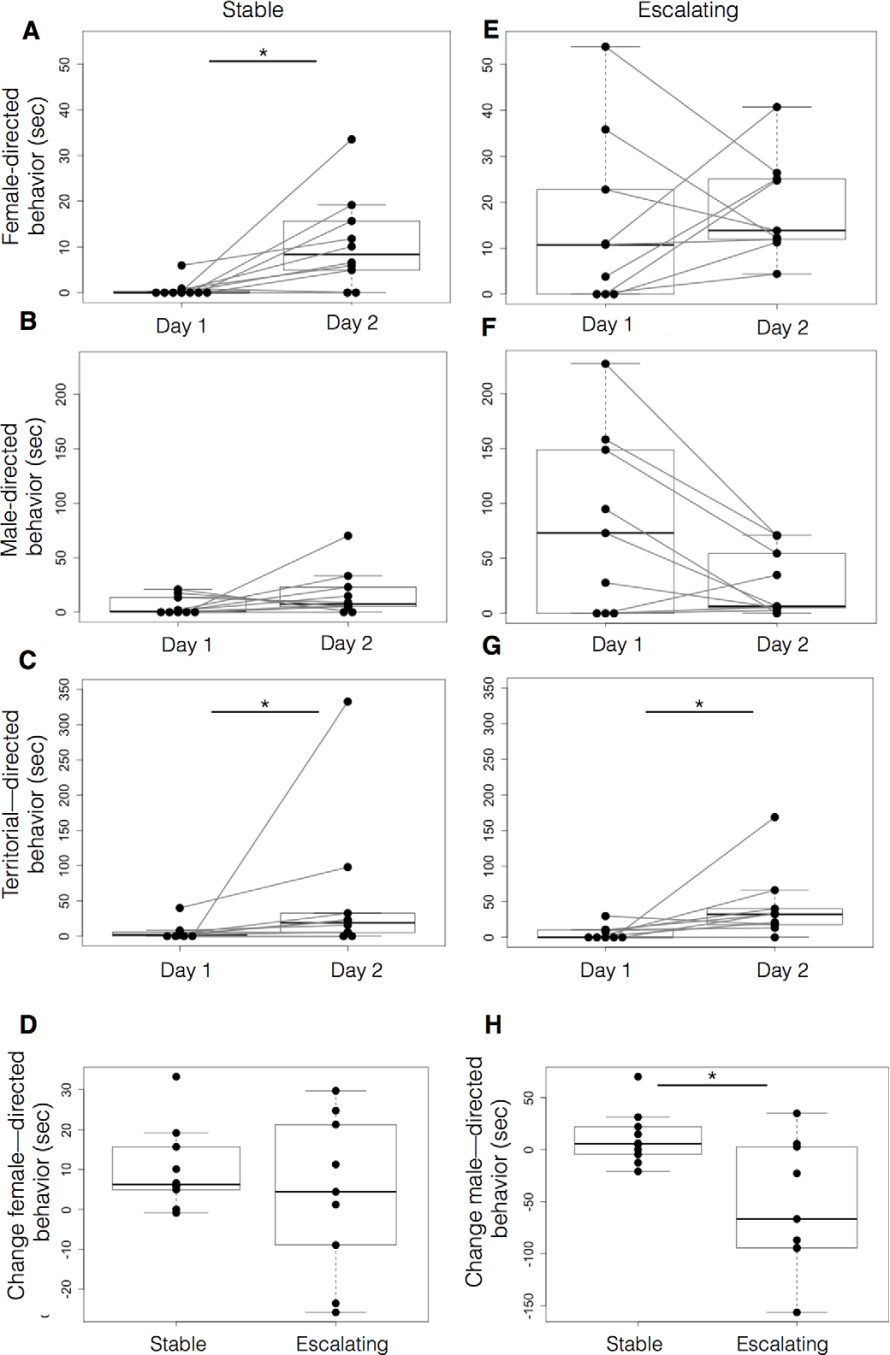
**Behavioral changes in stable and escalating aggressive males in a novel environment and one day later.** The behavior quantified for 15 min of observation on Day 1 and Day 2 shows the behavior of dominant males changes between Day 1 and Day 2 of the assay. On Day 1, escalating aggressive males displayed more male- and female-directed behaviors compared to the stable group (A). On Day 2 the behavior of the dominant male, clearly identified in each dyad, does not differ between the groups. We compared the female- and male-directed behaviors of each dominant fish on Day 2. Behaviors are expressed as time spent (s) engaged in either male- or female-directed behaviors. Data are plotted as mean time spent (s). Top and bottom of boxes represent the first and third quartiles, respectively; whiskers extend to the most extreme data points no more than 1.5 times the interquartile range from the box; and horizontal lines within the boxes represent group medians. Asterisks indicate a statistically significant difference (*P<0.05, Mann–Whitney).

To determine predictive patterns in male behavior prior to classification, we compared changes in behavior from Day 1 (new territory) to Day 2 (known territory) within groups (Fig. 5). We found that on Day 2 stable aggressive males increased the total time they spent performing female-directed behaviors (*P*=0.0059, Wilcoxon signed ranks; Fig. 5A) but not male-directed behaviors (*P*=0.1934, Fig. 5B). In contrast, in males with escalating aggression, female-directed behavior started at a higher level on Day 1 and did not change significantly from Day 1 to Day 2 (*P*=0.5703, Fig. 5E). Both groups performed more non-social territorial behaviors on Day 2 (Wilcoxon signed-ranks; escalating aggression, *P*=0.0098, Fig. 5G; stable aggression, *P*=0.0195, Fig. 5C), indicating that stable aggressive fish are active in non-social behaviors but increased their courtship behaviors only after securing a territory on Day 1.

Taken together, these data across Day 1 and Day 2 and across male types (stable versus escalating) suggest the novel environment did not affect the social behaviors of males with escalating aggression but did subdue the stable aggressive males' behaviors.

### Escalating aggressive males spent more time confronting a male intruder while stable males spent more time attending to females

On Day 16 we introduced a novel intruder into dyad tanks and observed the social behaviors of our established dominant males over the following 15 min. We found that escalating aggressive males engaged in more social interaction than stable aggressives (total social behaviors, *P*=0.0133, not shown; Mann–Whitney), and that this overall difference mostly consisted of directed encounters against the intruder male (*P*=0.0115, Fig. 3H), but not females (*P*=0.5936, Fig. 3G) or non-social territorial (*P*=0.116, Fig. 3I).

**Fig. 6.**
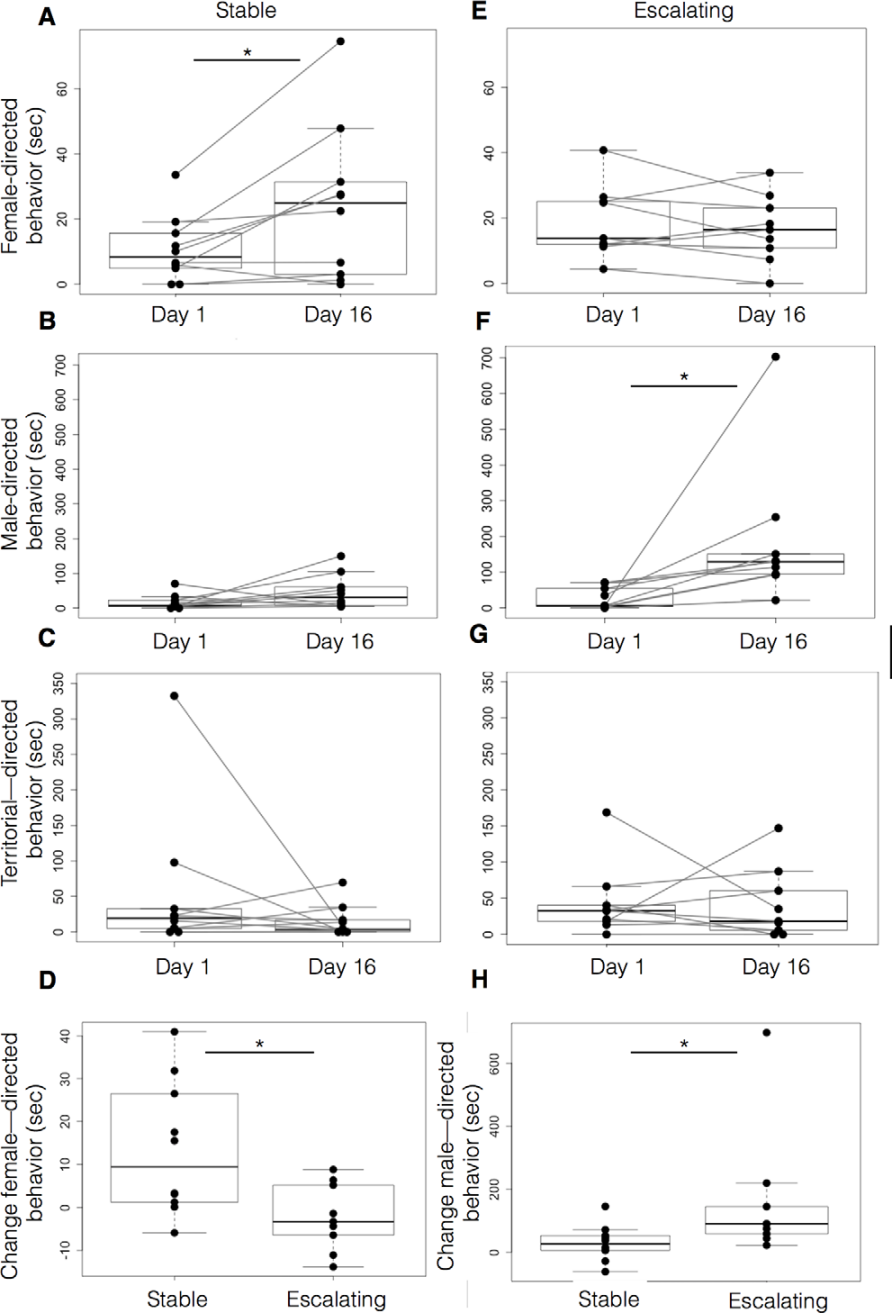
**Behavioral differences across social contexts between stable and escalating dominant males.** The behavior quantified for 15 min on Day 2 and Day 16 shows differences in behavior between dominant males. On Day 2 the dominant male, clearly identified in each dyad, directed its behavior towards females, opponent or territory. We compared the female- and male-directed behaviors of each dominant fish on Day 2 with the same behaviors on Day 16. When faced with an intruder, the stable subtype showed greater female-directed behaviors on Day 16 (C). In contrast, the escalating subtype performed more male-directed behaviors (E). We compared the relative magnitude of behavioral changes, expressed as the difference in seconds of total duration of behaviors, from Day 2 to Day 16 for the male types. There was no difference in non-social territorial behaviors. Differences in both female-directed behaviors of stable males, and male-directed in escalating males were significant (D,H). Behaviors are expressed as time spent (s) engaged in either male- or female-directed behaviors. Data are plotted as mean time spent (s). Top and bottom of boxes represent the first and third quartiles, respectively; whiskers extend to the most extreme data points no more than 1.5 times the interquartile range from the box; and horizontal lines within the boxes represent group medians. Asterisks indicate a statistically significant difference (*P<0.05, Mann–Whitney).

Next, we tested for changes in behavior within each type (Fig. 6), between Day 2 (when the opponent was familiar) and Day 16 (after introducing an intruder). We found that males with escalating aggression spent significantly more time performing social behaviors on Day 16 (Wilcoxon signed rank, *P*=0.0039, not shown). This change resulted from an increase in male-directed (*P*=0.0039, Wilcoxon signed ranks; Fig. 6F), but not female-directed (*P*=0.652, Fig. 6E) or territorial neutral (*P*=0.945, Fig. 6G) behaviors. By contrast, stable aggressive fish significantly increased female-directed (*P*=0.0195, Fig. 6A), but not male-directed (*P*=0.131, Fig. 6B) or territorial neutral (*P*=0.734, Fig. 6C) behaviors over the same time.

We also compared the change in time spent in female- and male-directed behaviors from Day 2 to Day 16 across escalating and stable aggression groups, and found that males with stable aggression increased their female-directed behavior significantly more than males with escalating aggression (*P*=0.027, Mann–Whitney; Fig. 6D), and increased their male-directed behavior significantly less than males with escalating aggression (*P*=0.017, Fig. 6H). There was no difference in territorial behaviors between stable and escalating aggressive males (*P*=0.959, not shown). Taken together, these data show that among established dominant males, those with escalating aggression tended to focus their attention on a novel challenger and spent more time attacking the intruder than males with stable aggression. In contrast, males with stable aggression spent increased amounts of time attending to females.

### Escalating aggressive males had lower baseline levels of estradiol and cortisol and greater hormonal responses of all steroid levels tested after an intruder challenge

**Fig. 7.**
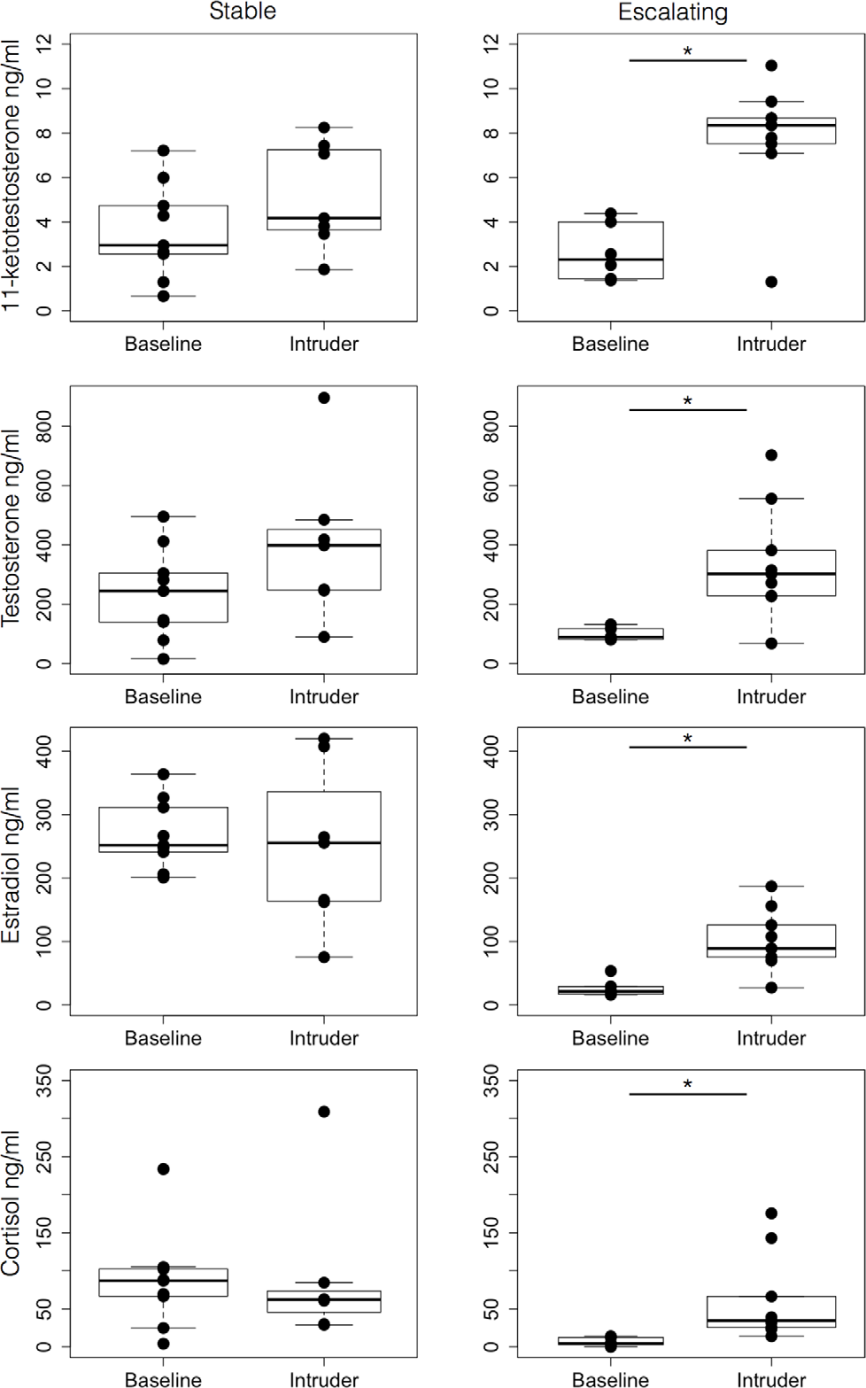
**Effect of an intruder male on circulating hormone levels of resident dominant males.** Circulating levels of 11-ketotestosterone, testosterone, estradiol and cortisol (rows) from a control group and a group exposed to an intruder challenge are shown for stable males (left column) and escalating males (right column). 11-ketotestosterone, testosterone, estradiol and cortisol levels increased significantly in escalating males when faced with an intruder, but no effect was observed in stable males. Data are plotted as concentration (ng/ml). Top and bottom of boxes represent the first and third quartiles, respectively; whiskers extend to the most extreme data points no more than 1.5 times the interquartile range from the box; and horizontal lines within the boxes represent group medians. Asterisks indicate a statistically significant difference (*P<0.05, Mann–Whitney).

To discover whether the differences in behavior correlated to differences in endocrine responses, we compared the hormone levels of stable and escalating males immediately after the 15 min intruder test, and again 9 days after the intruder test. We found no difference in baseline 11-KT levels between males with escalating aggression (*n*=6) and stable aggression (*n*=9, *P*=0.372, Mann–Whitney). We found lower levels of baseline estradiol (*P*=0.0004) and cortisol (*P*=0.0028) in the escalating males and a slight trend for lower testosterone (*P*=0.0867). Next, we compared hormone levels of males immediately after facing an intruder for 15 min, to males of the same class 9 days later (Fig. 7). When confronted with an intruder, the escalating aggressive males (*n*=9) showed circulating levels of 11-KT, testosterone, estradiol, and cortisol that were significantly higher than baseline levels (*n*=6; 11-KT, *P*=0.0120; testosterone, *P*=0.0120; estradiol, *P*=0.0016; cortisol, *P*=0.0004; Fig. 7 right). There was no such effect in the stable aggressive fish (Fig. 7, left). Finally, the peak level of 11-KT was significantly higher in escalating versus stable aggressive fish immediately after they faced an intruder (*P*=0.0311), while estradiol levels (*P*=0.0164) and cortisol levels were lower (*P*=0.0017). There was no significant difference for testosterone.

Thus, males with escalating aggression exhibited similar baseline levels of 11-KT and testosterone, and lower baseline levels of estradiol and cortisol than males with stable aggression. Furthermore, when challenged by an intruder, escalating aggressive males' hormone levels increased, especially in the case of 11-KT, which increased to a level significantly higher than that in stable aggressive fish.

## DISCUSSION

Dominant *A. burtoni* males have been typically classified as a uniform group and studied as a counterpart to subordinate males, each having distinct behavioral and physiological traits. *Post hoc* analysis of aggressive behavior in dyad males reared for other experiments revealed that some dominant males escalate their aggression over time while others remain relatively stable. These two types showed measurable differences in endocrine patterns and behaviors, which is defined as one of the principles for identifying personality types by Sih and Bell (2008). This study shows dominant males can be divided into two distinct sub-groups: males with escalating aggression that damage multiple fish over time, and males with stable aggression behaviorally defined as those that do not inflict damage on more than one fish. The persistence of these two types in the laboratory stock suggests individuals with distinctive styles of aggression are maintained in spite of laboratory selection and may represent polymorphisms present in the parental population (Wolf and Weissing, 2010). We used aggressivity to classify animals as a framework for examining the behaviors and physiology that distinguish these types of dominant fish over time, starting with the establishment of their territory and ending in a confrontation with a novel intruder.

In rodent studies, more aggressive individuals were found to be rigid in their behaviors and unaffected by changes in the physical environment in contrast to the less aggressive type, that modulate their behavior in a novel environment (Koolhaas et al., 1999). Similarly here, we found males exhibiting escalating aggression were more active socially, courting females and confronting an opponent soon after being placed in unfamiliar territory. In contrast, males with stable aggression took longer to display both aggressive behaviors towards males and courting behaviors towards females, suggesting these males may assess the novel environment and prepare strategies for attack and courtship before acting. We found no differences in the behavior of the subordinates that were paired with either type of male, suggesting the differences in behaviors are intrinsic to the dominant males and are not a response to the behavior of subordinates.

In a previous study (Alcazar et al., 2014) we found younger, less experienced males were quick to attack, similar to the escalating aggression class in this study, while the more experienced males were slow to attack, similar to the stable aggression class described here. Taken together, this suggests a mix of genetic and learned traits are responsible for differing modes of aggressive behavior in *A. burtoni*. It may also suggest individuals with escalating aggression do not learn to stabilize their responses with experience.

Data from Day 16, when males confronted an intruder in their established territory, show, through differing behavior, that the classes of dominant males persist over time. The escalating aggressive males spent tenfold more time attacking intruders than stable aggressive males. They also differed in their response to females, with the escalating aggressive males showing no change in their attention to females over time, versus the stable aggressive males, who increased their attention to females over time.

We tested four steroid hormones (estradiol, testosterone, 11-KT and cortisol) in animals immediately after intruder encounter and compared them between groups and to baseline levels (hormone levels measured later in a stable non-changing environment). Our findings are consistent with past research showing a relationship between steroid hormones and aggression in fish (Fernald, 1976; Hirschenhauser et al., 2004; Kime, 1993) and mammals (Trainor et al., 2007a,b, 2008). Specifically, males with escalating aggression have a response akin to that predicted by the ‘challenge hypothesis’ described by Wingfield et al. (1990) responding to intruders with higher levels not only of testosterone as shown by Wingfield but of all four steroid hormones tested in our study.

While O'Connell and Hofmann (2012) showed that circulating levels of steroids at the time of a challenge could be the underlying cause affecting the social status outcome of a fight, here we show that when dominant males are subdivided into escalating and stable aggressive types, the escalating aggressive males had low levels of baseline circulating steroids and summoned hormonal spikes during a fight, especially of 11-KT. Our study adds to our understanding of steroid response in *A. burtoni* males as it was previously shown that dominant males injected with testosterone show significantly increased male-directed aggression (Fernald, 1976). Moreover, inhibiting aromatase, the enzyme that converts testosterone to estradiol, decreases aggressive but not reproductive behaviors in dominant males (Huffman et al., 2013). This suggests reducing availability of estradiol reduces aggression. However the new findings offered by this study show that in the class of escalating aggressive dominant males it is not the level of estradiol (which is low) that is significant, but the spike. Mammalian studies also show that estrogen increases aggressive behavior. Within 15 min of injection of estradiol, mice show increased aggression in an intruder assay. This rapidity suggests the genes for this increase were not produced in response to the hormonal signal (there was no time for this) but immediately responded to already existing receptors (Trainor et al., 2008).

In *A. burtoni,* dominant and long-term suppressed subordinate males have significant differences in levels of steroid hormones (Fernald, 2012). Dominant males have higher levels of both testosterone and 11-KT [an androgen important in social interactions in teleost fish (Parikh et al., 2006)], as well as higher levels of estradiol than subordinates. Dominant males have lower levels of cortisol than subordinates and males that changed from dominant to subordinate status showed increases in cortisol levels (Fox et al., 1997). Additional studies indicate that during the transition from subordinate to dominant status, the circulating levels of androgens (testosterone, 11-KT), estradiol, and cortisol show marked increases within 30 min after the onset of social ascent (Maruska and Fernald, 2010a; Maruska et al., 2013), although once social status is stable, cortisol levels drop in dominant males. Given that *A. burtoni* has two androgen receptors (ARα and ARβ) and two estrogen receptors (ERα and ERβ) and that these receptors are different between dominant and subordinate types, there may be receptor differences among males that are predisposed to become more aggressive and it may be that these differences are a cause rather than a consequence of social status outcomes. Further studies of the strength and quality of receptors in these two subclasses of dominant males may lead to an understanding of how stable aggressive dominant males, whose baseline hormone levels are significantly higher than those of escalating aggressives, may be better capable of modulating their aggressive levels and adapting to the signals of the opponent.

As a candidate for the study of adaptive and maladaptive aggression, *A. burtoni* offers some intrinsic advantages. First, territorial contests for dominance are an ecologically valid stressor which elicits aggressive behavior and to which the species has been adapted. Second, as this study strongly suggests, among dominant males there is inter-individual variation in aggressive behavior extending to maladaptive aggression, e.g. greater damage to females. Third, the model developed by this study allows researchers to predict escalating versus stable aggression and therefore presents a means of testing methods of early intervention.

Stress-related disorders such as post-traumatic-stress-disorder (PTSD) are associated with dysregulation of the hypothalamus-pituitary-adrenal (HPA) axis (Horn et al., 2014). In some features, the phenotype of the male *A. burtoni* with escalating aggression resembles the characteristics of PTSD patients, including low baseline cortisol levels and spikes in times of stress. Since not all individuals that are exposed to trauma develop PTSD, the tendency to do so may be regulated by neuroendocrine responses to stress. Possible points of dysregulation of the HPA axis include the hypothalamus, pituitary, and adrenal glands (interrenal gland in fish), and the associated production and receptor mechanisms of the signaling molecules, CRF (cortico-releasing factor), ACTH (adrenocorticotropic hormone) and cortisol. Further investigations of these mechanisms may identify differences in the neural sensitivity to steroids between stable and escalating aggressive *A. burtoni* types, which may provide insights into how behavior, particularly aggression, is modulated by the neuroendocrine response (Daskalakis et al., 2013; Daskalakis and Yehuda, 2014).

## MATERIALS AND METHODS

### Animal care

*Astatotilapia burtoni*, derived from wild-caught stock (Fernald and Hirata, 1977a) were maintained in aquaria under conditions mimicking their natural habitat (28°C, pH 8, 12 h:12 h light:dark cycle, constant aeration and water chemistry matched to that of Lake Tanganyika). Fish were fed cichlid flakes (AquaDine, Healdsburg, CA, USA) and brine shrimp once a day, and reared in community tanks (∼35 fish per tank). Community tanks were 114 liters (91.4×55.9×30.5 cm, l×w×h) with four terracotta pots cut in half lengthwise to produce a truncated half cone (11×11×5.5 cm, l×w×h). Animals were kept at similar population densities across tanks to minimize differences in the number of social interactions (Fraley and Fernald, 1982) and in population growth rates since experience can bias winning outcomes (Alcazar et al., 2014). All of our experimental procedures were approved by the Stanford Administrative Panel for Laboratory Animal Care.

### Dominant-subordinate dyads

In the laboratory, all *A. burtoni* males will become dominant if their ownership of a territory is not challenged or if they win a territorial contest. A male will ascend to dominant status when no other male is present, including in complete isolation, suggesting that dominance is the default state of males, and that subordinate males are suppressed dominant males (Fernald, 1980), resulting from a dominance contest. Indeed, subordination requires the presence of a dominant. Experimentally, we generated 50% dominant males by pairing two males (dyads) with three females for up to 6 weeks in individual 30-liter tanks to create dominant-subordinate pairs. Since dominant status is the default state, subordinates are only created by constant exposure to aggressive behavior from the dominant male. Even males in isolation will perform dominant behavior and ultimately develop characteristically dominant physiology (Fernald, 1980). Table 1 summarizes the outcome of 318 dominant-subordinate dyads. We controlled for age since we previously showed that age can be an advantage in territorial contests between young adults (Alcazar et al., 2014). We restricted our experiments to males that were young adults (within 5-6 months of age), and chose animals that showed few territorial behaviors in rearing tanks to minimize differences in life-experience across competitors. Males accumulate behavioral differences from past winning and losing experiences. We matched males in age (within 1 week) and length (within 10%). We maintained rearing tanks with similar population densities to minimize differences in the number of social interactions over time. Suppression of the reproductive axis in subordinates is only obtained after 3-6 weeks of sustained social subordination (Fox et al., 1997). Dominant male aggression towards subordinates can be mild, consisting mostly of ritualized displays, but aggressive behavior of some dominant individuals can escalate to chasing, biting, and ramming.

We initially paired males to identify dominant and subordinate individuals, keeping track of the number of fish damaged and the time between introducing the challenger and the damage. In cases where the dominant male showed high aggression, as defined by evidence of damage to fins and/or other body parts in the subordinate, we removed the subordinate and introduced a second male. This would typically result in another fight between the resident dominant male and the intruder to determine which male would become dominant. Males were matched in size and age, so individuals that were residents (familiar with the territory) in every case won the fight against the intruder. In most cases, if the dominant male attacked the newly introduced second male, the dominant male was sacrificed and tissue and blood were collected. However, occasionally the second damaged male was removed and we introduced a third male. If the third male also showed signs of body damage from attacks by the dominant resident male, then the dominant male was removed, sacrificed and tissue samples were collected. All males that we report as damaging three or more fish (three females were present with all dyads) had also damaged females. In all dyads, if females showed body injuries they were promptly removed and exchanged for new females. Each morning, animals were assessed for injury and those that appeared damaged were removed and replaced. We sacrificed and measured the standard length and weight and collected brain, pituitary, testes, and plasma for hormone measurements of all dominant males. The large population available for the *post hoc* analysis presented here used the dyads generated for numerous undergraduate projects and other studies (Alcazar et al., 2014; Brawand et al., 2014).

### Longitudinal assay

The classifications of ‘stable’ and ‘escalating’ aggressive fish were made at the end of the assay during data analysis, so the observer cataloging behavior was blind as to which class a given fish would be assigned. We recorded male behavior during three 15-min periods (Day 1, Day 2, Day 16) to identify the relationship of a dominant's aggression class and his behavior during his first territory establishment (Day 1) and during an intruder assay (Day 16). The first recording (Day 1) took place immediately after transferring two males and three females from rearing tanks to a test tank (no signs of exaggerated aggression were detectable at this point). These males had not previously experienced a dyad set up, and had no previous experience with their opponent. We matched competitors for age and size (length within 3%). A second recording (Day 2) was taken at the same time of the day, the following day. The third recording (Day 16) took place two weeks after Day 2. During the intervening time damaged fish were replaced. Replacement fish were of the same age and size as the established dominant male. On Day 16, subordinates were removed in the morning, a male intruder was added to the test tank in the afternoon (3-4 h later) and the behavior of the resident dominant male was recorded immediately. All intruder assays were done at the same time in the afternoon to avoid differences related to time of day. We marked animals by clipping the dorsal fin uniquely. As in all dominant-subordinate dyad assay set-ups, each morning dyads were observed and if females or the subordinate male showed damage, they were replaced with new animals. For all dominant males, upon sacrifice, we measured the standard length, weight and collected brain, pituitary, testes, and plasma for hormone measurements.

An observer, blind to the experimental conditions, scored 15-min videos of Day 1, Day 2 and Day 16 using a specialized behavioral observation program (Wu et al., 2009). The observer scored the following behaviors using categories identified by Fernald and Hirata (1977a).

- Male-directed: chases, flares fins, bumps, lateral displays, approaches opponent, quivers to male, frontal threat;
- Female-directed: chases, quivers to female, leads female to spawn;
- Territorial neutral: digs and removes gravel from pot, pot entries and exits;
- Total social behaviors: combination of male- and female-directed behaviors.

### Hormone measurements for both studies above

Dominant males collected in both assays were measured for standard length (±1 mm), weighed (±0.001 g), and blood samples were taken by caudal severance into 100 μl capillary tubes within 2 min of capture. Blood samples were centrifuged for 10 min (5939×***g***) and plasma removed and stored at −80°C until assayed. Testes were also removed, weighed, and gonadosomatic index calculated [GSI=(gonad weight/body weight×100)]. Plasma samples (4-28 μl) were reconstituted in assay buffer depending on the assay [cortisol (Cort; 1:50-400), testosterone (T; 1:400-1600), 11-ketotestosterone (11KT; 1:500), estradiol (E_2_; 1:1250)] prior to analysis. We followed the protocol for enzyme-linked immunoabsorbant assays (ELISA). Kits used are the following: Cortisol EIA kit (#500360), Estradiol EIA kit (#582251), Testosterone EIA kit (#582701) and 11KT EIA kit (#582751) (Cayman Chemical, Inc.). All samples were assayed in duplicate, plates were read at 405 nm using a microplate reader (UV Microplate Reader, Molecular Devices), and hormone levels were determined by standard curve. Intra-assay coefficients of variation (CV) were: Cort (10.1%, 18.9%); T (4.5%, 9.4%); 11-KT (6.7%, 10.6%); and E_2_ (10.6%, 17.28%, 13.8%). For samples compared across plates, E_2_ (plates with intra-assay CV of 10.6% and 17.28%) inter-assay CV was 15.2%. Plasma samples of stable and escalating aggressive males were distributed equally between plates to minimize inter-assay effects. Assay validation was previously established for *A. burtoni* samples (e.g. Parikh et al., 2006; Maruska and Fernald, 2010b).

### Statistics and clustering

All statistical tests were performed in the R statistical computing environment or Prism 6.0. The Mann–Whitney U test (aka Wilcoxon rank-sum) was used to assess possible effects between two independent groups. The Kruskal–Wallis extension of Mann–Whitney was used when there were more than two independent groups. The Wilcoxon signed-ranks test was used to assess possible effects across time-points, i.e. in situations with repeated measures.

To cluster animals based on their Day 1 dyad behaviors we first computed all pairwise Pearson correlations between animals based on the number of times they performed different behaviors (see above) on that day. Two animals that did not perform any behaviors were necessarily excluded from this part of the analysis. The study used 1−correlation as the input distance metric to the R function *hclust*. Average linkages were used to build the hierarchical dendrogram in Fig. 3, and a dynamic tree-cutting algorithm (Langfelder et al., 2008) was used to define distinct clusters in an unsupervised fashion. Three animals were not closely associated enough with other samples to be assigned to a cluster. Chi-squared tests were used to quantify the likelihood of randomly observing the distribution of escalating and stable aggression animals across clusters that we saw. When a test is specified in the text all subsequent *P*-values were computed using that same test until otherwise noted.
